# Correlation between motor function and health-related quality of life in early to mid-stage patients with Parkinson disease: a cross-sectional observational study

**DOI:** 10.3389/fnagi.2024.1399285

**Published:** 2024-06-24

**Authors:** Ying Ge, Wowa Zhao, Lu Zhang, Xiaoyi Zhao, Xuan Shu, Jiawei Li, Ying Liu

**Affiliations:** Department of Rehabilitation Medicine, Peking Union Medical College Hospital, Chinese Academy of Medical Sciences, Beijing, China

**Keywords:** Parkinson’s disease, motor function, health-related quality of life, balance, gait

## Abstract

**Aim:**

To investigate the correlation between motor function and health-related quality of life (HrQOL) in early to mid-stage patients with Parkinson disease (PwP).

**Methods:**

This cross-sectional study recruited PwP from April 2020 to December 2023 at the outpatient clinic of Peking Union Medical College Hospital in Beijing, China. The motor symptoms were assessed using Movement Disorder Society–sponsored revision of the Unified Parkinson’s Disease Rating Scale (MDS-UPDRS) part 3. Balance function was evaluated using the Berg Balance Scale (BBS), and the risk of fall using Timed Up-and-Go test (TUG), and Five Times Sit-to-Stand test (FTSST). Freezing of gait questionnaire (FOGQ) was used to evaluate the severity of gait. The Intelligent Device for Energy Expenditure and Physical Activity (IDEEA) recorded gait cycle parameters, and the isokinetic dynamometer measured muscle strength. The Parkinson’s Disease Questionnaire-39 (PDQ-39) was used to measure HrQOL. All assessments were tested during the on state. Spearman correlation was conducted to evaluate the correlation between motor function and HrQOL.

**Results:**

243 patients with mean age of 69.33 years were enrolled. The PDQ-39 score was strongly correlated with FOG in H&Y stage III (*r* = 0.653, *p* < 0.001) and moderately correlated in H&Y stage I (*r* = 0.471, *p* < 0.001) and H&Y stage II (*r* = 0.386, *p* < 0.001). Furthermore, the FOG was strongly correlated with mobility domain at H&Y stage III (*r* = 0.694, *p* < 0.001) and moderately correlated at H&Y stage I (*r* = 0.431, *p* < 0.001) and H&Y stage II (*r* = 0.434, *p* < 0.001). All motor function scores were correlated with PDQ-39 scores at H&Y stage III (*p* < 0.05).

**Conclusion:**

Motor function correlated with HrQOL in early to mid-stage PwP, and FOG was the main factor, especially affecting mobility, activities of daily life and communication. HrQOL in patients at different disease stages were variously affected by motor function, and HrQOL and multiple dimensions was significantly associated with motor function in patients at H&Y stage III.

## Introduction

Parkinson’s disease (PD) is a chronic progressive neurodegenerative disorder characterized by both motor and non-motor symptoms (Bloem et al., 2021). The prevalence of PD is increasing among elderly patients in China (Qi et al., 2021). The main motor features are resting tremor, bradykinesia, rigidity, shuffling gait, and postural instability, which have negative impacts on health-related quality of life (HrQOL) of PwP (Beitz, 2014). Early in the course of the disease, symptoms are usually mild, the drug response is usually reliable and the patient functions well. As the disease progresses, there is an increasing impact on and dependence on activities of daily living (Sveinbjornsdottir, 2016).

There is an increasing clinical focus on HrQOL which provides a multi-dimensional view of the patient’s well-being, and a guiding principle for researchers and clinicians (Bock et al., 2022). Strong evidence indicates that motor and non-motor symptoms in PwP limit their independence, essential activities of daily living and social participation. Impairment in these domains can alter social roles by affecting employment status, home management, friendships, and other relationships. Conversely, a decline in HrQOL can have a negative impact on motor and non-motor symptoms (Galeoto et al., 2022).

Previous studies (Gomez-Esteban et al., 2007; Kurihara et al., 2020; Bock et al., 2022) have demonstrated a significant negative correlation between motor symptoms and HrQOL. However, their assessments were self-reported and did not specifically explore which aspect of motor symptoms had the determinant impact on HrQOL at different disease stages. Scalzo et al. (2012) recruited thirty-six patients and found that the impairment in balance while performing functional activities and the reduction in walking capacity negatively affect the perception of HrQOL. But the study had some limitation such as small sample size and incomplete assessment of motor function. There are numerous studies (San Martín Valenzuela et al., 2020; Szefler-Derela et al., 2020; Li et al., 2021) showing improvements in motor function and quality of life through rehabilitation. However, there is still a lack of consensus on how to implement more effective rehabilitation training programs. Therefore, this observational cross-sectional study aims to investigate the relationship between motor function and HrQOL across different disease severity and hypothesizes that balance function and risk of falling would have the greatest impact on HrQOL.

## Methods

### Study design and participants

This cross-sectional observational study was approved by the Ethics Committee and Institutional Review Board of the Peking Union Medical College Hospital and written informed consent was obtained from each patient before beginning the study.

The inclusion criteria were as follows: (a) diagnosis of primary Parkinson’s disease according to the Movement Disorder Society Clinical Diagnostic Criteria for Parkinson’s disease, (b) Hoehn and Yahr (H&Y) stages I-III, (c) stable medication regimen for at least 4 weeks before the study. Exclusion criteria included (a) atypical Parkinson’s syndrome, (b) severe nervous system disease other than PD or motor system disease, (c) severe cognitive disorder, or mental disorder, (d) severe visual or hearing impairment, (e) poor compliance, (f) refusal to sign the written informed consent.

### Clinical assessments

First, patients completed the demographic questionnaire, including patients’ information, height, weight, BMI (weight in kilograms divided by the square of height in meters). Then the balance function, risk of fall, FOGQ, MDS-UPDRS, lower extremity muscle strength and HrQOL were evaluated, respectively. H&Y stages and drug On-phase were provided by a neurologist and all assessments were performed in 1 day and in the drug On-phase by a physical therapist.

### Health related quality of life

HrQOL was ascertained by the PDQ-39 (The 39-item Parkinson’s disease questionnaire), a tool for the systematic assessment of the impact of PD on an individual’s quality of life. It is a well-validated measure using 5 levels of severity to measure health status of 8 domains, including mobility (10 items), activities of daily living (6 items), emotional well-being (6 items), stigma (4 items), social support (3 items), cognitions (4 items), communication (3 items) and bodily discomfort (3 items) (Jenkinson et al., 1997). The higher scores indicate lower levels of quality of life. The Chinese version PDQ-39 was proved a reliable and valid measure in both research and clinical trials (Zhang and Chan, 2012).

### Motion function assessment

#### Balance

The Berg balance scale (BBS) is a 14-item test, using scores from 0 to 4 for each item, designed to measure static and dynamic standing balance. The total score ranges from 0 to 56, with higher scores indicating better balance, and a score of less than 46 out of 56 indicates the presence of risk falls (Alagumoorthi et al., 2022).

#### Risk of fall

The timed up-and-go test (TUG), and the five times sit to stand test (FTSST) were used to evaluate the risk of fall of PwP. The TUG Test measured the time in which participants take to rise from the chair, walk at a normal, self-selected pace to a line marked on a hardwood floor three meters from the start line, turn around, walk back to the chair and sit down (Nightingale et al., 2019). The proposed cut-off score of 11.5 s was suggested for discriminating between PwP who did and did not fall (Nocera et al., 2013). The FTSST measured the time in which participants take to stand up and sit down as quickly as possible for five repetitions (Goldberg et al., 2012). The proposed cut-off score for discrimination between PwP who did and did not fall was 16.0 s (Duncan et al., 2011).

#### Gait cycle

Temporal–spatial gait parameters of PD were acquired using IDEEA activity monitor (Minisun, Fresno, CA) (de la Cámara et al., 2019). Participants completed four flat 10 m walking trials with the device on as required by the test. The data collected on stride length, stride speed and foot angle off the ground are used as the main indicators to assess the PD patient’s walking function.

#### Isokinetic muscle strength test

Knee flexor and extensor isokinetic peak torque were evaluated using the isokinetic dynamometer (ISOMOVE, Tecnobody Company, Italy) (Hasan et al., 2021). Participants were seated on the dynamometer, and the belts were used at the thigh, pelvis, and trunk to avoid compensatory movements. The testing procedure consisted of respective five knee flexor and extensor contractions. Participants were asked to perform the movement with their maximal strength.

### Movement disorder society–sponsored revision of the unified Parkinson’s disease rating scale

The MDS-UPDRS is the most widely used clinical scoring system to measure the severity and progression of PD (Martinez-Martin et al., 2015). MDS-UPDRS Part III evaluates 18 motor examination items by the rater (Ueno et al., 2020). Each item is scored from 0 to 4, with higher scores indicating poorer motor function.

### Statistical analysis

Summary statistics, including means and standard deviations for continuous variables, and counts and percentages for categorical data, were employed for variable description. The Spearman correlation coefficient *r* was used to evaluate the correlation between PDQ-39 and motor function. Correlations were deemed weak when coefficients were < 0.30, moderate from 0.30 to 0.59, and strong >0.60 (Yoon et al., 2017). The significance level was set at *p* = 0.05. Statistical analyses were performed with SPSS 25.0 (SPSS Inc., Chicago, IL, United States).

## Results

### Participant characteristics

243 PD patients, aged 60 to 80 years old, admitted to the department of rehabilitation in Peking Union Medical College Hospital from April 2020 to December 2023 were recruited. Patients had a mean age 69.33 years (SD = 6.61), with 45.7% being women. Demographic and clinical characteristics of PwP at different H&Y stages are shown in Table 1.

**Table 1:** Demographic and clinical features of patients with Parkinson’s disease among different H&Y stages (PD) (*n* = 243).

Variables [Mean ± SD or *n* (%)]	H&Y stage I	H&Y stage II	H&Y stage III
*n* (%)	87 (35.80%)	80 (32.92%)	76 (31.27%)
Age (yrs.)	66.26 ± 6.17	70.56 ± 6.14	71.53 ± 6.33
Female	37	57	38
	50	23	38
BMI	23.84 ± 3	23.16 ± 2.6	23.71 ± 3.09
MDS-UPDRS Part III	13.75 ± 5.46	24.74 ± 7.39	29.5 ± 9.49
Balance and Risk of Fall			
BBS	51.47 ± 3.19	46.23 ± 3.86	38.39 ± 7.29
TUG (seconds)	11.44 ± 18.4	13.09 ± 4.75	20.97 ± 20.61
FTSST (seconds)	11.35 ± 2.79	14.05 ± 4.71	17.25 ± 8.12
Muscle strength outcomes			
Knee flexion (N·m)	24.86 ± 12.31	18.92 ± 9.14	13.51 ± 7.51
Knee extension (N·m)	50.68 ± 22.36	41.18 ± 19.54	29.14 ± 15.95
Gait cycle assessment			
FOG	4.25 ± 4.32	7.21 ± 5.79	9.72 ± 6.97
Step length(m)	0.51 ± 0.07	0.45 ± 0.09	0.38 ± 0.1
Speed of gait(m/s)	0.96 ± 0.18	0.82 ± 0.17	0.67 ± 0.2
Toe-off angle (°)	30.97 ± 11.57	24.09 ± 10.82	21.07 ± 8.64
Quality of life			
Mobility	12.16 ± 15.31	26.53 ± 18.78	39.08 ± 26.43
Activities of daily living	10.35 ± 11.57	23.39 ± 20.59	28.34 ± 25.76
Emotional well-being	17.67 ± 17.07	24.9 ± 20.97	16.89 ± 19.85
Stigma	19.83 ± 22.52	28.59 ± 28.05	22.53 ± 23.39
Social support	8.81 ± 14.84	14.27 ± 18	11.4 ± 17.52
Cognition	24.07 ± 16.33	27.97 ± 18.28	29.03 ± 21.3
Communication	8.14 ± 14.49	16.87 ± 19.12	27.3 ± 28.01
Bodily discomfort	28.83 ± 20.95	31.77 ± 21.95	37.94 ± 25.76
PDQ-39 SI	16.23 ± 10.77	24.29 ± 13.34	26.56 ± 15.51

BMI, body mass index; H&Y, Hoehn and Yahr; MDS-UPDRS, movement disorder society–sponsored revision of the Unified Parkinson’s Disease Rating Scale; BBS, Berg Balance Scale; TUG, timed up-to-go test; FTSST, five times sit-to-stand test; FOG, freezing of gait; PDQ-39 SI, the 39-item Parkinson’s disease questionnaire summary index score.

### Correlation

Correlation analyses were conducted to evaluate different association of motor function and HrQOL at different H&Y stages (Table 2). The PDQ-39 score was strongly correlated with FOG at H&Y stage III (*r* = 0.653, *p* < 0.001) and moderately correlated at H&Y stage I (*r* = 0.471, *p* < 0.001) and H&Y stage II (*r* = 0.386, *p* < 0.001). Additionally, the PDQ-39 score was significantly correlated with MDS-UPDRS Part III, TUG, step length and toe off angle at H&Y stage I, and BBS, TUG and FTSST at H&Y stage II. All motor function scores were correlated with PDQ-39 scores at H&Y stage III (*p* < 0.05). This study found that age and BMI had no evident correlation with PDQ-39 SI (*p* < 0.05).

**Table 2:** Coefficient of correlation of Spearman (*r*) and *p*-value between age, motor function and PDQ-39 among different H&Y stages.

Variables	H&Y Stage I		H&Y Stage II		H&Y Stage III	
	*r*	*p*	*r*	*p*	*r*	*p*
Age	0.162	0.133	−0.22	0.850	−0.083	0.476
BMI	−0.034	0.756	0.007	0.850	−0.054	0.640
MDS-UPDRS Part III	0.252*	0.019	0.181	0.109	0.619*	<0.001
BBS	−0.088	0.416	−0.333*	0.003	−0.489*	<0.001
TUG (seconds)	0.238*	0.036	0.302*	0.006	0.312*	0.006
FTSST (seconds)	0.156	0.148	0.301*	0.007	0.255*	0.027
Knee flexion strength (N·m)	−0.182	0.092	−0.205	0.068	−0.359*	<0.001
Knee extension strength (N·m)	−0.145	0.180	−0.218	0.052	−0.399*	<0.001
FOG	0.471*	<0.001	0.386*	<0.001	0.653*	<0.001
Step length (m)	−0.226*	0.035	−0.182	0.107	−0.449*	<0.001
Speed of gait (m/s)	−0.183	0.090	−0.146	0.196	−0.346*	0.002
Toe-off angle (°)	−0.227*	0.034	0.209	0.062	−0.238*	0.038

BMI, body mass index; MDS-UPDRS, movement disorder society–sponsored revision of the Unified Parkinson’s Disease Rating Scale; BBS, Berg Balance Scale; TUG, timed up-to-go test; FTSST, five times sit-to-stand test; FOG, freezing of gait. *Indicates a significant correlation between age, motor function and PDQ-39.

Then, the correlation between motor function and the 8 dimensions of PDQ-39 was further analyzed (Table 3). The FOG was strongly correlated with mobility at H&Y stage III (*r* = 0.694, *p* < 0.001) and moderately correlated at H&Y stage I (*r* = 0.431, *p* < 0.001) and H&Y stage II (*r* = 0.434, *p* < 0.001). And mobility was correlated with all motor function assessment scores at H&Y stage III (*p* < 0.001), with FOG and BBS score being the most correlated factors. UPDRS Part III, FOG and BBS were the most correlated factors of activities of daily life at H&Y stage II & III (*p* < 0.001). Furthermore, communication was strongly correlated with UPDRS Part III and FOG at H&Y stage III (*p* < 0.001).

**Table 3:** Coefficient of correlation of Spearman between motor function and dimensions of PDQ-39.

	H&Y stage	Mobility		Activities of daily living		Emotional well-being		Stigma		Social support		Cognition		Communication		Bodily discomfort	
		r	*p*	r	*p*	r	*p*	r	*p*	r	*p*	r	*p*	r	*p*	r	*p*
UPDRS Part III	I	0.294*	0.006	0.237*	0.027	0.075	0.489	0.152	0.159	0.061	0.574	0.083	0.443	0.147	0.176	0.264*	0.013
	II	0.389*	<0.001	0.314*	0.005	0.087	0.441	0.02	0.859	−0.119	0.295	0.096	0.398	0.302*	0.006	0.03	0.792
	III	0.543*	<0.001	0.649*	<0.001	0.296*	0.009	0.129	0.266	0.131	0.261	0.430*	<0.001	0.546*	<0.001	0.141	0.225
BBS	I	−0.124	0.251	0.007	0.946	−0.088	0.418	0.071	0.511	−0.011	0.918	0.084	0.442	0.060	0.578	−0.172	0.111
	II	−0.496*	<0.001	−0.395*	<0.001	−0.091	0.423	−0.221*	0.049	0.087	0.44	−0.181	0.109	−0.242*	0.03	−0.188	0.095
	III	−0.644*	<0.001	−0.611*	<0.001	−0.290*	0.011	−0.159	0.171	−0.008	0.948	−0.225	0.051	−0.352*	0.002	−0.14	0.228
TUG (seconds)	I	0.252*	0.019	0.161	0.136	0.185	0.086	0.078	0.472	0.134	0.215	0.037	0.735	0.318*	0.003	0.156	0.149
	II	0.407*	<0.001	0.291*	0.009	0.036	0.754	0.171	0.13	−0.095	0.403	0.141	0.212	0.218	0.052	0.201	0.073
	III	0.446*	<0.001	0.340*	0.003	0.232*	0.044	0.109	0.347	0.128	0.269	0.033	0.774	0.472*	<0.001	0.127	0.274
FTSST (seconds)	I	0.166	0.124	0.093	0.393	0.143	0.186	0.101	0.354	0.021	0.847	−0.014	0.897	0.238*	0.026	0.182	0.091
	II	0.185	0.101	0.250*	0.025	0.167	0.14	0.287*	0.01	0.027	0.815	0.039	0.731	0.284*	0.011	0.294*	0.008
	III	0.279*	0.015	0.129	0.269	0.223	0.054	0.197	0.09	0.141	0.228	0.083	0.481	0.292*	0.011	0.144	0.218
Knee flexion (N·m)	I	−0.174	0.107	−0.148	0.171	0.007	0.951	−0.205	0.057	−0.103	0.343	0.008	0.944	0.035	0.745	−0.238*	0.026
	II	−0.349*	0.002	−0.237*	0.035	−0.109	0.335	−0.118	0.296	0.117	0.303	−0.07	0.539	−0.196	0.081	−0.059	0.6
	III	−0.385*	0.001	−0.443*	<0.001	−0.260*	0.023	−0.239*	0.038	−0.284*	0.013	0.055	0.637	−0.298*	0.009	−0.162	0.162
Knee extension (N·m)	I	−0.088	0.420	−0.142	0.188	0.048	0.662	−0.108	0.318	<0.001	0.998	−0.125	0.248	0.050	0.644	−0.239*	0.026
	II	−0.441*	<0.001	−0.243*	0.03	−0.037	0.742	−0.114	0.313	−0.001	0.99	−0.074	0.515	−0.099	0.384	−0.152	0.178
	III	−0.337*	0.003	−0.386*	0.001	−0.263*	0.022	−0.236*	0.04	−0.257*	0.025	−0.17	0.142	−0.289*	0.011	−0.240*	0.036
FOG	I	0.431*	<0.001	0.199	0.065	0.357*	0.001	0.143	0.187	0.419*	<0.001	0.253*	0.018	0.21	0.051	0.350*	0.001
	II	0.434*	<0.001	0.329*	0.003	0.188	0.095	0.179	0.112	0.208	0.064	0.213	0.058	0.173	0.125	0.349*	0.002
	III	0.694*	<0.001	0.639*	<0.001	0.312*	0.006	0.197	0.087	0.034	0.773	0.336*	0.003	0.511*	<0.001	0.406*	<0.001
Step length (m)	I	−0.099	0.363	−0.187	0.082	−0.245*	0.022	−0.072	0.506	−0.126	0.246	−0.031	0.773	−0.078	0.473	−0.165	0.128
	II	−0.519*	<0.001	−0.265*	0.018	−0.047	0.676	−0.044	0.701	0.003	0.982	−0.079	0.485	−0.023	0.839	−0.081	0.478
	III	−0.612*	<0.001	−0.417*	<0.001	−0.347*	0.002	−0.079	0.498	−0.106	0.364	−0.123	0.291	−0.366*	0.001	−0.295*	0.01
Speed of gait (m/s)	I	−0.128	0.239	−0.148	0.17	−0.195	0.071	−0.106	0.328	−0.093	0.39	0.022	0.84	−0.156	0.15	−0.109	0.313
	II	−0.437*	<0.001	−0.166	0.142	−0.029	0.8	−0.001	0.993	0.059	0.602	−0.111	0.329	−0.01	0.929	−0.083	0.462
	III	−0.489*	<0.001	−0.282*	0.014	−0.289*	0.011	−0.046	0.694	−0.12	0.302	0.043	0.713	−0.257*	0.025	−0.268*	0.019
Toe-off angle (°)	I	−0.177	0.102	−0.19	0.078	−0.220*	0.041	−0.08	0.463	−0.225*	0.036	−0.124	0.252	−0.307*	0.004	0.002	0.986
	II	−0.305*	0.006	−0.173	0.126	−0.015	0.898	−0.182	0.106	−0.033	0.769	−0.07	0.54	−0.167	0.138	−0.071	0.531
	III	−0.311*	0.006	−0.174	0.132	−0.196	0.089	−0.036	0.757	0.024	0.836	−0.128	0.271	−0.155	0.182	−0.109	0.349

MDS-UPDRS, movement disorder society–sponsored revision of the Unified Parkinson’s Disease Rating Scale; BBS, Berg Balance Scale; TUG, timed up-to-go test; FTSST, five times sit-to-stand test; FOG, freezing of gait. *Indicates a significant correlation between motor function and dimensions of PDQ-39.

## Discussion

PD is a progressive multi-system neurodegenerative disease affecting people mainly in later years of life. Treatment should focus on functional capacity and quality of life throughout the entire course of disease (Barbosa et al., 2022). However, rehabilitation training is likely to have a more obvious impact in the early to mid-stage of the disease and may prevent or slow down further functional decline (Kluger et al., 2014). This study aims to identify the most impaired motor function caused by the disease and the most relevant HrQOL factors, and then provide a target for rehabilitation training to improve HrQOL in early to mid-stage PD.

FOG is a unique and disabling clinical phenomenon characterized by brief episodes of inability to step or by extremely short steps that typically occur on initiating gait or on turning while walking. Physiological, functional imaging, and clinical pathological studies point to disturbances in frontal cortical regions, the basal ganglia, and the midbrain locomotor region as the probable origins of FOG (Gao et al., 2020). FOG is very common in PD and one of the most relevant features of PD closely related to falls (Bloem et al., 2004; Bekkers et al., 2018; Zhang et al., 2021). Frequent falls lead to injury, fear of falling, reduced mobility and social isolation (Thanvi and Treadwell, 2010). Mehdizadeh et al. (2016) showed that regardless of balance impairments, fear of fall has a negative association with HRQOL in PwP. This study showed that FOG was correlated with HrQOL at H&Y stages I ~ III,which accord with the finding of Ellis et al. (2011) and Nakano et al. (2021). Further analysis showed FOG was correlated with mobility, ADL, and communication. Previous study (Cosentino et al., 2020) proved that physical therapy may improve FOG, for instance, action observation, treadmill combined with cueing, and prolonged home-based exercise trainings. In this study, step length and speed of gait have moderate correlation and toe-off angle had weak correlation with PDQ-39 SI at H&Y stages III, which mainly affected mobility domain. In rehabilitation practice, targeted training, e.g., LSVT BIG (Flood et al., 2020) can be made to address these gait problems of patients.

This study found the moderate association between balance function and HrQOL at H&Y stages II& III, but with no significant association at H&Y stages I. This study shows that patients at H&Y stage I have the highest BBS score and little or mild balance impairment, which is not significantly associated with their HrQOL. Poor balance will lead to poor HrQOL (Nakano et al., 2021), which was found in patients at H&Y stages II& III in this study. Meanwhile, Scalzo et al. (2012) found that patients with severe balance dysfunctions assessed by the BBS and who scored lower distances in the 6 min walking test had worse HrQOL. Furthermore, longer time of TUG and FTSST led to poor HrQOL, which means that high risk of fall negatively affected HrQOL. According to the further analysis, BBS, TUG and FTSST mainly affected mobility and ADL domains. Bailey et al. (2022) also found that PwP who performed better on the 4 m walk speed test, the standing balance test, had higher scores on the total PDQ-39, mobility, ADL, cognition and bodily discomfort domains. Conventional understanding suggests that patients at H&Y stage II do not experience balance symptoms, but this study showed that their balance function had deteriorated and negatively affected their HrQOL. Therefore, balance training should be considered earlier in clinical practice.

Muscle strength in PwP is impaired compared with healthy controls (Skinner et al., 2019; Renee et al., 2021), with the largest deficits observed in the lower extremity in the “OFF” medication state and of concentric contractions (Gamborg et al., 2023). Yokote et al. (2022) found that lower muscle strength correlates with gait performance in advanced Parkinson Disease. As a meta-analysis (Hart et al., 2023) reported that the prevalence of probable sarcopenia, a condition associated with the loss of skeletal muscle strength and mass, ranged from 23.9–66.7% in the PwP population. This study showed moderately correlation between knee strength and HrQOL in patients at H&Y stages III. Therefore, muscle strength training, especially in patients at H&Y stages III may improve in gait performance and HrQOL and prevent the presence of sarcopenia.

This study found that motor function correlated with HrQOL especially in mobility and activities of life. The most interesting finding of the current study showed that motor function has strong correlation with communication at H&Y stages III. Communication impairment is common in PD and may be due to motor language control and cognitive-linguistic disorder (Smith and Caplan, 2018). PDQ-39 has one question on speech and two question on communication with other people. Motor function assessments represent disease severity and the relationship with motor language control or cognitive-linguistic disorder should be further explored.

Strengths of the study include the identification of FOG as the most important factor affecting HRQOL, and the differential association between motor function and HRQOL in patients at different H&Y stages, supported by a robust sample size. Additionally, the potential correlation between motor function and communication function warrants further investigation. However, this study has some limitations. This analysis was cross-sectional and therefore conclusions were restricted to one point in time. Therefore, further studies should be done to investigate whether increasing or decreasing motion function results in HrQOL changes in PwP. In the future, there will be a need for a larger and longer cohort.

## Conclusion

In conclusion, motor function correlated with HrQOL in early to mid-stages PwP, and FOG was the main factor, especially in mobility, activities of daily life and communication. In addition, HrQOL was correlated with gait and risk of fall at H&Y stage I, and gait, balance, and risk of fall at H&Y stages II. All motor function were associated with HrQOL and multiple dimensions in patients at H&Y stage III. Therefore, Physical therapy should be targeted according to disease stages to obtain the most improvement of HrQOL.

## Data availability statement

The raw data supporting the conclusions of this article will be made available by the authors, without undue reservation.

## Ethics statement

The studies involving humans were approved by the Ethics Committee and Institutional Review Board of the Peking Union Medical College Hospital. The studies were conducted in accordance with the local legislation and institutional requirements. The participants provided their written informed consent to participate in this study.

## Author contributions

YG: Formal analysis, Writing – original draft. WZ: Investigation, Writing – review & editing. LZ: Project administration, Methodology, Writing – review & editing. XZ: Supervision, Writing – review & editing. XS: Writing – review & editing. JL: Writing – review & editing. YL: Conceptualization, Funding acquisition, Writing – review & editing.
